# Drebrin Regulates Acetylcholine Receptor Clustering and Organization of Microtubules at the Postsynaptic Machinery

**DOI:** 10.3390/ijms22179387

**Published:** 2021-08-30

**Authors:** Paloma Alvarez-Suarez, Natalia Nowak, Anna Protasiuk-Filipunas, Hiroyuki Yamazaki, Tomasz J. Prószyński, Marta Gawor

**Affiliations:** 1 Nencki Institute of Experimental Biology, Polish Academy of Sciences, 3 Pasteur Street, 02-093 Warsaw, Poland; p.alvarez-suarez@nencki.edu.pl (P.A.-S.); n.nowak@nencki.edu.pl (N.N.); annaprotasiuk@yahoo.pl (A.P.-F.); Tomasz.Proszynski@port.lukasiewicz.gov.pl (T.J.P.); 2 Gunma University Graduate School of Medicine, Maebashi, Gunma 371-8511, Japan; spikar@gunma-u.ac.jp

**Keywords:** drebrin, EB3, rapsyn, AChR clustering, actin, microtubules, cytoskeleton, neuromuscular junction, postsynaptic machinery

## Abstract

Proper muscle function depends on the neuromuscular junctions (NMJs), which mature postnatally to complex “pretzel-like” structures, allowing for effective synaptic transmission. Postsynaptic acetylcholine receptors (AChRs) at NMJs are anchored in the actin cytoskeleton and clustered by the scaffold protein rapsyn, recruiting various actin-organizing proteins. Mechanisms driving the maturation of the postsynaptic machinery and regulating rapsyn interactions with the cytoskeleton are still poorly understood. Drebrin is an actin and microtubule cross-linker essential for the functioning of the synapses in the brain, but its role at NMJs remains elusive. We used immunohistochemistry, RNA interference, drebrin inhibitor 3,5-bis-trifluoromethyl pyrazole (BTP2) and co-immunopreciptation to explore the role of this protein at the postsynaptic machinery. We identify drebrin as a postsynaptic protein colocalizing with the AChRs both in vitro and in vivo. We also show that drebrin is enriched at synaptic podosomes. Downregulation of drebrin or blocking its interaction with actin in cultured myotubes impairs the organization of AChR clusters and the cluster-associated microtubule network. Finally, we demonstrate that drebrin interacts with rapsyn and a drebrin interactor, plus-end-tracking protein EB3. Our results reveal an interplay between drebrin and cluster-stabilizing machinery involving rapsyn, actin cytoskeleton, and microtubules.

## 1. Introduction

The synapses formed between motoneurons and muscle fibers, termed neuromuscular junctions (NMJs) are responsible for the transmission of signals from the nerve to the muscle, thus enabling crucial functions of the organism, such as movement or respiration [1,2]. The vertebrate neuromuscular junctions constitute a specialized ‘tripartite system’ composed of presynaptic motoneuron terminal, perisynaptic Schwann cells, and postsynaptic apparatus on the muscle fiber [1,3,4]. NMJs are formed before birth as plaques and they mature postnatally to acquire a complex topology [5,6,7,8]. In mice, NMJs undergo intensive remodeling starting around P7 up to adulthood (around P30) and during that process, they transform from simple oval plaques into ‘pretzel-like’ structures. The changes in the acetylcholine receptor (AChR) organization are accompanied by the major reshaping of the cortical cytoskeleton [8,9]. Structural transformation of the NMJ into an elaborate, perforated AChR array mirroring the shape of the nerve terminal is pivotal for efficient synaptic transmission [10]. Importantly, disrupted NMJ morphology was reported in various myopathies, such as Duchenne muscular dystrophy, myasthenia gravis, congenital myasthenic syndromes, and spinal muscular atrophy [11,12,13,14,15,16]. Despite the importance of NMJ maturation, little is known about molecular mechanisms underpinning this process. It is postulated that dynamic actin-rich structures, termed synaptic podosomes, remodel the maturing postsynaptic machinery in cultured myotubes and contribute to the formation of the perforations between AChRs [17,18,19]. When podosome formation is pharmacologically inhibited in vitro, the morphology of AChR clusters in myotubes is disrupted [17]. However, the potential role of synaptic podosomes at NMJs still requires verification.

Pre- and postsynaptic compartments of the NMJ communicate with each other through synaptic proteins, which participate in the signal transduction and connect different NMJ domains to the extracellular matrix (ECM) and cytoskeleton [1,8,20]. Postsynaptic proteins are anchored to the actin filaments and microtubules (MTs) by the dystrophin-glycoprotein complex (DGC), focal adhesion proteins, and the scaffold protein rapsyn, which is essential for AChR clustering [21,22,23,24,25]. The finely tuned interplay between the membrane-associated actin cytoskeleton and microtubules controls the dynamics of AChRs at the postsynaptic sarcolemma [25,26]. Remodeling of the actin cytoskeleton drives AChR trafficking and aggregation into clusters [26,27]. One of the key regulators of the AChR clustering is the Rho family of GTPases, which interact with actin-binding proteins, thus controlling local assembly or disassembly of actin filaments [28,29,30,31,32]. We have recently shown that ArhGef5, a Guanine nucleotide-exchange factor (GEF) for Rho GTPases, is involved in NMJ stability [33]. Moreover, in vitro maturation of AChR clusters is accompanied by the formation of synaptic podosomes, rich in actin and various actin-binding proteins, such as Arp2, Tks5, cortactin, dynamin, filamin, and angiomotin-like 2 (Amotl2) [18,34,35]. Knockdown of Amotl2, a member of the angiomotin family, impacts both organization of the AChR clusters and the structure of synaptic podosomes in myotubes [18]. Recent studies showed that microtubule-asssociated GTPase, dynamin2, is essential for podosome and cluster perforation dynamics and the organization of postsynaptic cytoskeleton at Drosophila NMJs [35]. Apart from the actin cytoskeleton, the high-density specialized microtubule network plays an important role in the postsynaptic machinery [36,37]. MTs control the distribution of AChRs at the postsynaptic membrane by guiding their transport and incorporation into clusters [37,38,39,40]. The targeting of dynamic MTs into the postsynaptic specialization is led by plus-end-tracking proteins (+TIP), such as CLASP2 [37,41]. The function of microtubules is regulated by their post-translational modifications, nevertheless, how microtubules contribute to the organization of NMJs is a complex question that still remains open [40]. Despite the fact that cytoskeletal rearrangements govern the formation, maturation, and maintenance of NMJs, the underlying molecular mechanism is not fully understood [8,42].

Recent studies shed light on the role of proteins that are able to cross-link actin and microtubules in the NMJ maturation. The microtubule-actin cross-linking factor 1 (MACF1) was identified as a postsynaptic protein at NMJs [25]. MACF1 binds rapsyn and organizes actin cytoskeleton and MTs through the interaction with the actin-binding protein vinculin and EB1, which belongs to +TIPs [25]. Conditional knockout of MACF1 results in morphological and functional impairments of the neuromuscular synapses [25,43]. Importantly, mutations in the *MACF1* gene were also identified in patients suffering from congenital myasthenia [25]. Another co-organizer of actin and MTs is drebrin, a protein extensively studied at the synapses of the central nervous system (CNS). Drebrin is abundant at the postsynaptic cytoskeleton of dendritic spines and has a major role in the development of CNS, dendritic spine formation, and synaptic plasticity [44,45,46,47,48,49,50]. Moreover, drebrin has been identified at the cell junctions and podosomes in various cell types and the localization of drebrin was reported in motoneurons [51,52,53,54,55]. In neurons, drebrin targets microtubule plus-end protein EB3, involved in MT capture and delivery of AChRs to the postsynaptic sarcolemma at NMJs [37,38,56].

Despite its apparent synaptic role, drebrin function in skeletal muscle has been studied so far only in the context of myogenesis and myogenic differentiation, and the role of this protein at NMJs remains unclear [57]. In this paper, we aim to explore the role of drebrin in the organization of the postsynaptic machinery. We identify drebrin as a protein localized to the AChR domain of NMJs and to the postsynaptic machinery of cultured myotubes. Drebrin also colocalizes with podosomes formed during postsynaptic maturation. We show that drebrin downregulation or inhibition of its interaction with actin impairs the targeting of microtubules to AChR clusters and disrupts microtubule organization at the postsynaptic machinery in vitro. Finally, we show that both drebrin and EB3 bind rapsyn, a key regulator of the AChR clustering. Our results highlight novel mechanisms, which regulate postsynaptic machinery formation and development, and might contribute to the better understanding of pathogenesis of myopathies.

## 2. Results

### 2.1. Drebrin Localizes to the AChR Compartment of Neuromuscular Junctions and to the Contractile Machinery of Muscle Sarcomeres

**Figure 1:**
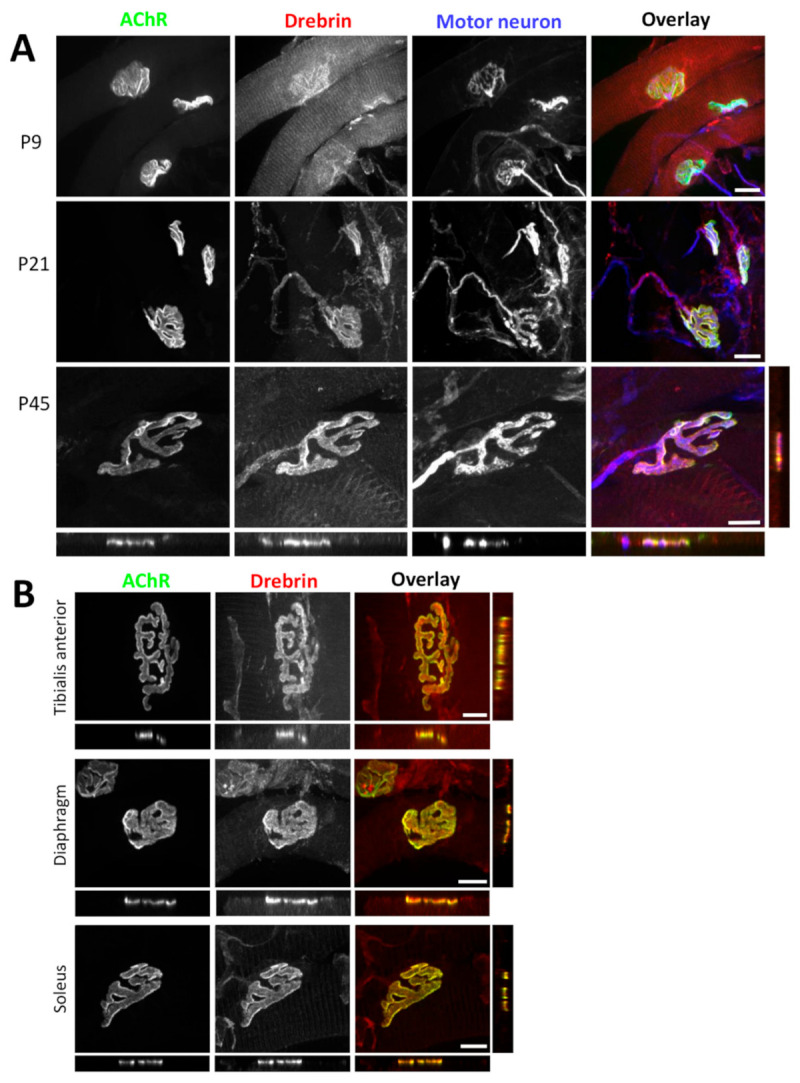
Drebrin localizes to the neuromuscular junction pre- and postsynaptic machinery. (**A**) Colocalization of drebrin (red) with bungarotoxin-labeled (BTX) acetylcholine receptor (AChR) clusters (green) and motoneuron (blue) in mouse *triangularis sterni* muscle during different postnatal stages (P9, P21, and P45). Bottom and right panels show an orthogonal view of P45 with visible colocalization of drebrin and AChRs. (**B**) Colocalization of drebrin (red) with AChRs (green) in fast-twitch (*tibialis anterior*, *diaphragm*) and slow-twitch (*soleus*) muscle types at P30. Bottom and right panels show orthogonal views. Scale bars—10 μm.

To test whether drebrin will be recruited to different structural domains during NMJ remodeling, we explored the localization of this protein in *triangularis sterni* muscle at P9, P21, and P45 with a special emphasis on the postsynaptic machinery, labeled with fluorescent bungarotoxin (BTX) that specifically binds to AChRs. We found similar drebrin localization patterns both at juvenile and adult NMJs, which overlapped with AChRs (Figure 1A). A thorough analysis of the image optical cross-sections revealed postsynaptic localization of drebrin, which colocalized with AChRs (Figure 1A, bottom and right panels). In line with previous studies, drebrin was also concentrated at the presynaptic domain, overlapping with synaptophysin and neurofilament markers (Figure 1A).

The properties of NMJs can be affected by the fiber type they innervate and result in differential response to stimuli depending on the metabolic properties, fatigue resistance, and mechanical responses [58]. Innervation, synapse morphology, and NMJ molecular machinery also vary between fiber types [59,60,61]. Taking this into consideration, we compared drebrin localization at NMJs of the muscles with different functions and fiber type compositions. We analyzed the localization of drebrin at NMJs of the *soleus* muscle, with predominantly slow fibers, and *diaphragm*, with predominantly fast fibers [62,63,64]. We also analyzed *tibialis anterior*, a predominantly fast-twitch muscle that has a considerable proportion (~25%) of intermediate fibers [65]. We found that drebrin localization at the NMJs did not depend on the fiber type and was enriched along the postsynaptic AChRs in all muscles analyzed (Figure 1B).

**Figure 2:**
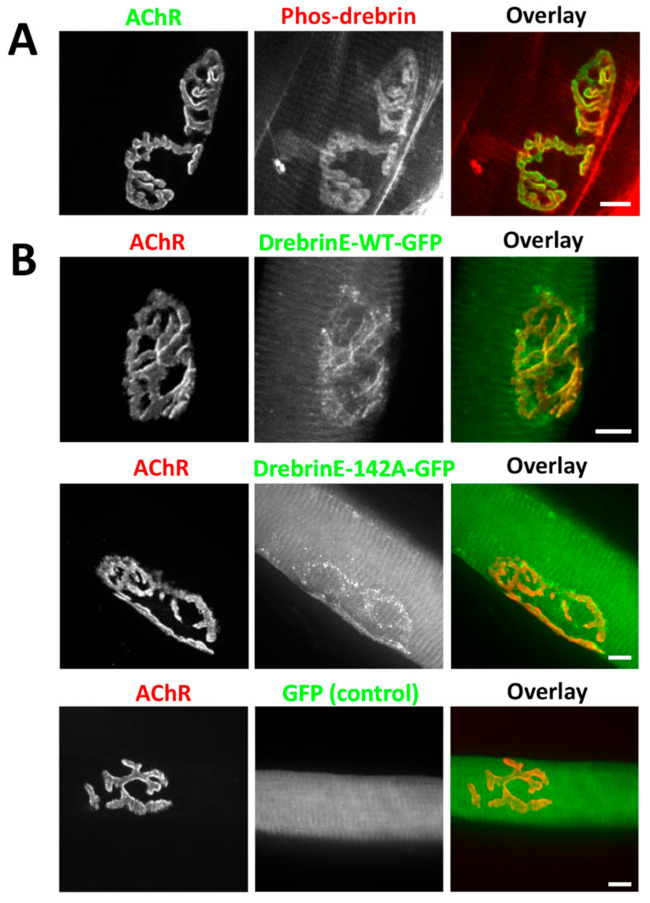
Phosphorylation does not affect drebrin localization at neuromuscular junctions (NMJs). (**A**) Colocalization of endogenous drebrin phosphorylated at Ser142 (red) with BTX-labeled AChRs (green) in *tibialis anterior* muscle. (**B**) Localization of exogenous wild-type drebrin (DrebrinE-WT-GFP, green), and phospho-dead mutant at Ser142 (DrebrinE-142A-GFP, green) overlaps with AChRs (red) at the NMJs of *tibialis anterior* muscle. Enhanced green fluorescent protein (EGFP) concentration at the NMJ (control) was not detected. Scale bars—10 μm.

Next, we assessed the specific localization of post-translationally modified drebrin at NMJs. Phosphorylation at Ser142 by Cdk5 was shown previously to regulate drebrin interaction with a +TIP family protein, EB3, and the recruitment of drebrin to MTs, which are crucial for the organization of AChRs at NMJs [37,38,66,67]. We used a phos-S142-specific antibody to check the localization pattern of phosphorylated drebrin at the adult NMJs. We found that phosphorylated drebrin was enriched at the AChR-rich NMJ branches, similarly to the localization pattern shown by the pan-drebrin antibody (Figure 2A).

Our findings were independently tested with the overexpression of different variants of drebrin E, which is a predominant isoform in myogenic cells [57]. We electroporated *tibialis anterior* with constructs encoding wild-type drebrinE-GFP and drebrinE-142A-GFP, a phospho-dead mutant in S142 residue. We found that both wild-type drebrinE and phospho-dead mutant drebrinE-142A were concentrated at NMJs and contractile machinery of muscle fibers (Figure 2B). The localization of wild-type and non-phosphorylatable drebrinE was overlapping with AChRs with a more dot-like pattern for the phospho-dead mutant (Figure 2B). No fluorescent signal enrichment was detected at NMJs after overexpression of enhanced green fluorescent protein (EGFP) (Figure 2B). These findings confirmed that drebrin is enriched at the AChR-rich domain of NMJs and showed that drebrinE phosphorylation at S142A site is dispensable for the recruitment of this protein to the AChRs. Altogether, our results show that drebrin is a new actin-organizing protein residing at the postsynaptic machinery of NMJs throughout postnatal development.

Simultaneously with the enrichment at NMJs, we observed that drebrin localizes in a striated pattern at the muscle fiber contractile machinery (Figure 1 and Figure 2). Drebrin is an important cytoskeletal organizer, hence we analyzed the colocalization of this protein with the markers specific to different contractile machinery domains, namely myosin heavy chain for thick filaments, Ryr1 for sarcoplasmic reticulum, and α-actinin for Z-lines [68,69,70]. In accordance with its actin-binding function, we observed that drebrin colocalizes with α-actinin, a protein that connects actin fibers to Z-lines (Supplementary Figure S1, bottom panel). Concomitantly, drebrin was in close vicinity, but not overlapping with, Ryr1 and myosin (Supplementary Figure S1). Thus, we conclude that together with α-actinin, drebrin is another actin-organizing protein present at Z-lines.

### 2.2. Drebrin Localizes to the Postsynaptic Machinery In Vitro

**Figure 3:**
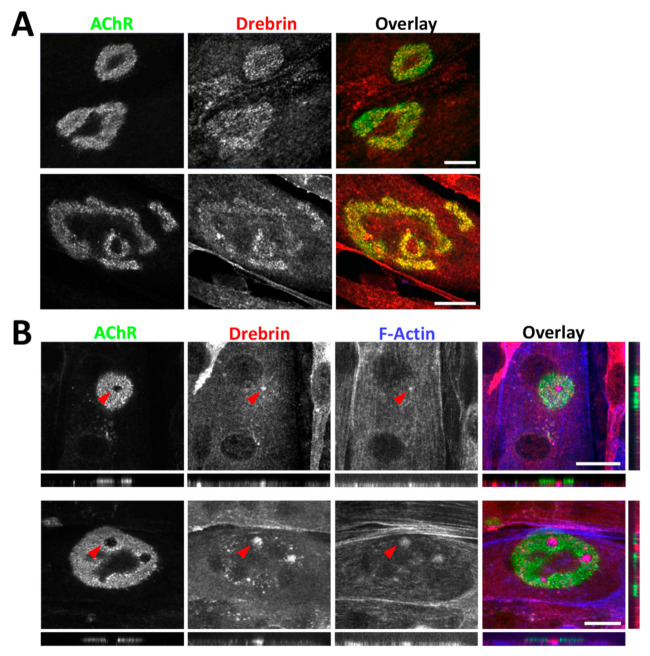
Drebrin localizes to the postsynaptic machinery in vitro. (**A**) Colocalization of drebrin (red) with BTX-labeled AChR clusters (green) in C2C12 cultured myotubes. (**B**) Colocalization of drebrin (red) with F-actin (blue) of synaptic podosomes in the perforations of simple (upper panel) and complex (bottom panel) AChR clusters (green). Red arrows indicate synaptic podosomes. The localization at synaptic podosomes was confirmed with two anti-drebrin antibodies: upper panel—10260-1-AP, Proteintech; lower panel—25770-1-AP, Proteintech. Bottom and right small panels show optical cross sections of the perforation area (arrow) with visible enrichment of drebrin. Scale bars—20 μm.

Next, we analyzed the localization of drebrin at the postsynaptic machinery in vitro. To induce the formation of AChR clusters, we used C2C12 myotubes plated on a laminin-111 substratum, allowing for the maturation of complex clusters resembling the NMJ topology [6,71]. Similar to NMJs, drebrin colocalized with AChRs and, additionally, was detected within the cluster perforations (Figure 3A). Previous studies have shown that perforations in the AChR clusters are occupied by the actin-rich organelles termed as synaptic podosomes, responsible for cluster remodeling [17]. Numerous cytoskeletal proteins were identified in the core of synaptic podosomes, such as beta-actin, Arp2, Amotl2, cortactin, and myosin IIA [34]. We tested drebrin localization at synaptic podosomes with two different antibodies and found that drebrin colocalizes with condensed F-actin in the perforations within AChR clusters (Figure 3B). The enrichment of drebrin was not limited to the surface of the perforation but was visible in the whole synaptic podosome located underneath (Figure 3B, panels show optical cross-sections of the perforation area marked by the red arrow). Therefore, our results shine a new light on drebrin as a potential regulator of the postsynaptic machinery organization and maturation in vitro.

### 2.3. Drebrin Downregulation or Impairment of Drebrin Binding to F-Actin Compromises AChR Clustering In Vitro

**Figure 4:**
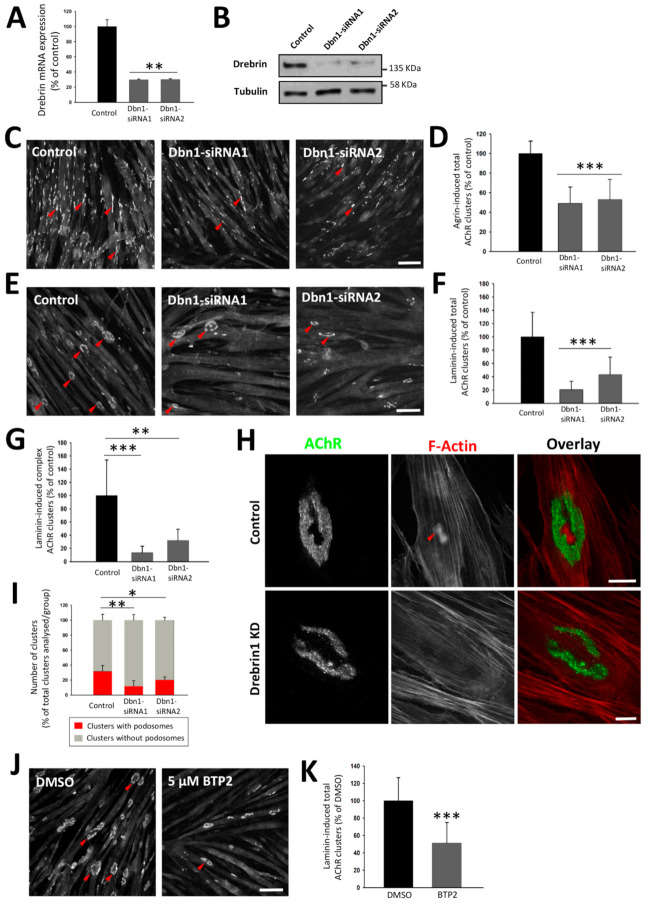
siRNA-mediated drebrin downregulation and inhibition of drebrin binding to F-actin with 3,5-bis-trifluoromethyl pyrazole (BTP2) impair AChR clustering in vitro. (**A**,**B**) Drebrin1 knockdown validation with RT-qPCR (**A**) and Western blot (**B**). (**C**) Agrin-induced AChR clusters labeled with BTX in C2C12 myotubes transfected with Dbn1-siRNAs. Red arrows indicate AChR clusters. (**D**) Quantification of the total number of agrin-induced AChR clusters in drebrin-depleted myotubes. (**E**) Laminin-induced AChR clusters labeled with BTX in C2C12 myotubes transfected with Dbn1-siRNAs. Red arrows indicate AChR clusters. Scale bars—100 μm. (**F**) Quantification of the total number of laminin-induced AChR clusters in drebrin-depleted myotubes. (**G**) Quantification of the number of laminin-induced complex AChR clusters in drebrin-depleted myotubes. Data are presented as a percentage of control (myotubes transfected with non-targeting siRNA). (**H**) Representative images of laminin-induced AChR clusters (green) and F-actin (red) in C2C12 myotubes transfected with Dbn1-siRNA2. Red arrow indicates synaptic podosomes. Scale bars—20 μm. (**I**) Quantification of the number of AChR clusters with synaptic podosomes in drebrin-depleted myotubes. Data are presented as a percentage of total clusters in each group. Error bars represent SD. Student’s *T* test, *p* ≤ 0.05 (*); *p* ≤ 0.01 (**); *p* ≤ 0.001 (***). (**J**) BTX-labeled AChR clusters (red arrows) in BTP2-treated and control myotubes incubated with dimethyl sulfoxide (DMSO) (vehicle). Scale bar—100 μm. (**K**) Quantification of the total number of laminin-induced AChR clusters in BTP2-treated and control myotubes.

Cytoskeletal organizers were previously shown to impact postsynaptic machinery organization [18,25,35,72]. We found that drebrin colocalizes with AChRs and synaptic podosomes involved in postsynaptic maturation. Therefore, we decided to investigate whether drebrin plays a role in the AChR cluster organization, and for that purpose, we used siRNA-mediated knockdown of *Dbn1* expression in cultured C2C12 myotubes. To induce the formation of AChR clusters, we applied two methods routinely used in the in vitro studies of the postsynaptic machinery: treatment of myotubes with soluble agrin or culturing them on laminin-111-coated surface. Laminin enables cluster maturation when simple plaques transform to perforated complex AChR assemblies resembling NMJ topology [6]. The Dbn1-siRNA transfection was performed at the stage when mature myotubes were already formed, which excludes the possibility that the disrupted AChR clustering was caused by the drebrin’s role in myoblast fusion [57]. The efficiency of the knockdown was evaluated at the mRNA and protein levels (Figure 4A,B and Supplementary Figure S2A). When AChR cluster formation was stimulated with agrin, drebrin downregulation decreased the number of AChR clusters approximately by half in comparison to myotubes transfected with control non-targeting siRNA (Figure 4C,D). In myotubes cultured on laminin-111, the negative effect on the formation of clusters was stronger, and the number of total and complex clusters was significantly reduced (Figure 4E–G and Supplementary Figure S2B). Since we detected drebrin at synaptic podosomes, we also examined whether drebrin knockdown could affect their formation. Indeed, the number of active actin-rich podosomes decreased in myotubes transfected with Dbn1-siRNAs in comparison to control (Figure 4H,I and Supplementary Figure S2C).

To further confirm that the observed phenotype is a result of impaired drebrin-mediated cytoskeletal rearrangements, we incubated myotubes with 3,5-bis-trifluoromethyl pyrazole (BTP2), a store-operated calcium channel inhibitor, which was previously shown to block the interaction between drebrin and F-actin [73]. BTP2 prevents C2C12 myotube formation when added up to 60 h after fusion induction [57]. To avoid such effects, BTP2 was added to myotubes 72 h after fusion when mature myotubes were already formed. Similarly to drebrin knockdown, we observed a significant reduction in the total number of AChR clusters (Figure 4J,K). Therefore, we found that drebrin interaction with actin is important for postsynaptic machinery clustering and maturation in vitro.

### 2.4. Drebrin Regulates Microtubule Capture at AChR Clusters

**Figure 5:**
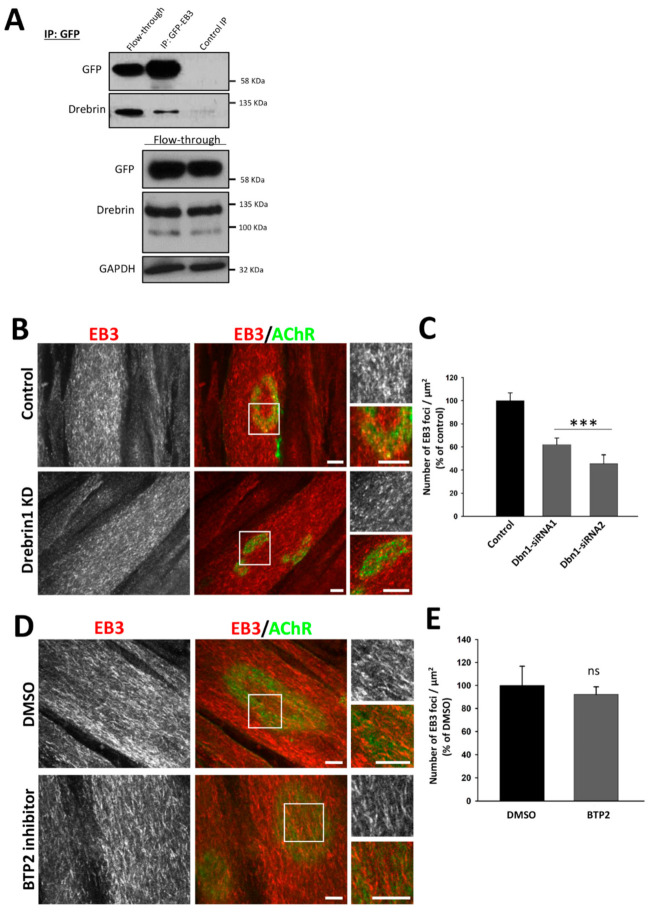
Drebrin and microtubule-associated EB3 interact in myotubes and drebrin knockdown impacts microtubule capture at AChR clusters. (**A**) Co-immunoprecipitation of endogenous drebrin with overexpressed EB3-GFP in homogenates from C2C12 myotubes cultured on laminin-111. Flow-through—lysate incubated with the beads, IP—eluate from the beads coated with anti-GFP antibody, Control IP—eluate from the uncoated beads. (**B**) Control and drebrin-depleted C2C12 myotubes labeled for AChRs (green) and microtubules plus-end protein EB3 (red). Representative images from control and drebrin knockdown with siRNA1 are shown. Control myotubes were transfected with non-targeting siRNA. Scale bars—10 μm. (**C**) Quantification of EB3 foci localized underneath AChR clusters after drebrin knockdown. (**D**) EB3 foci under AChR clusters in control and BTP2-treated C2C12 myotubes labeled for microtubules plus-end protein EB3 (red) and AChRs (green). Scale bars—10 μm. (**E**) Number of EB3 foci localized underneath AChR clusters upon BTP2 treatment. Data are presented as a percentage of control (myotubes transfected with non-targeting siRNA or myotubes incubated with DMSO (vehicle)). Error bars represent SEM. Student’s *T*-test, *p* ≤ 0.001 (***); ns—not significant.

Microtubules making contacts with AChRs were shown to play an important role in the assembly of postsynaptic machinery, especially in the incorporation of new AChRs to the existing clusters [37,38,39]. Importantly, drebrin organizes the microtubule network by interacting with the microtubule plus-end-tracking protein EB3 [66,67]. We decided to test whether impairment of the AChR organization was caused by the disrupted attachment of cluster-associated microtubules. First, we performed a co-immunoprecipitation assay to confirm the interaction of drebrin and a plus-end-binding protein EB3 in myotubes (Figure 5A). For this purpose, we used the SleepingBeauty transposon system [74] and created a C2C12 stable line that constitutively expressed GFP-fused EB3. Myoblasts were further differentiated into myotubes and AChR cluster formation was induced with laminin-111. Endogenous drebrin co-immunoprecipitated with exogenous EB3-GFP (Figure 5A), demonstrating that these two proteins interact in the cell line model that we used. Then, we performed immunostaining of microtubule plus-end marker EB3 and analyzed its distribution under AChR clusters in drebrin-depleted C2C12 myotubes (Figure 5B). We found that silencing of drebrin expression reduced the number of EB3 foci underneath AChR clusters compared to control myotubes (Figure 5C).

When we blocked drebrin binding to F-actin with BTP2, the number of EB3 foci under the AChRs remained similar to control (Figure 5E), however, some morphological differences in EB3-positive puncta organization were apparent (Figure 5D).

**Figure 6:**
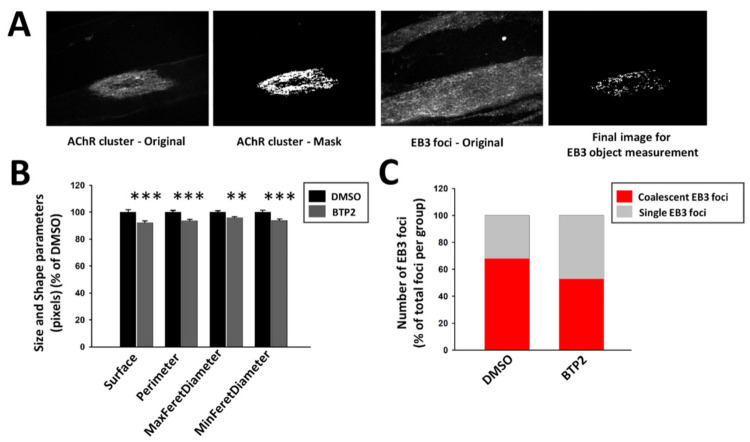
BTP2 impairs the organization of AChR cluster-associated mictrotubules. (**A**) Images used for processing in Ilastik and CellProfiler—segmentation and object identification. The last image on the right is an example of images used for object size and shape measurements (see Materials and Methods, Section 4.3). (**B**) Automated measurement of size and shape parameters of AChR cluster-associated EB3 foci after BTP2 treatment (CellProfiler). (**C**) Quantification of the total number of coalescent vs. single EB3 foci upon BTP2 treatment based on the surface manual measurements with ZEN software. Average surface of a single foci was used as a threshold and EB3 puncta with bigger surface were classified as coalescent. The distribution of coalescent EB3 foci is significantly different from the uniform distribution in both groups. Data are presented as a percentage of control (myotubes incubated with DMSO (vehicle)) except in (**C**), which represents a percentage of total EB3 foci analyzed in each group. Error bars represent SEM. (**B**) Student’s *T*-test, *p* ≤ 0.01 (**); *p* ≤ 0.001 (***); (**C**) chi-square test, *p* < 0.001, chi square = 681.727, 4 degrees of freedom.

The microtubule network is very dense in myotubes and difficult to analyze, so to overcome that we used automated analysis of the EB3-positive puncta combining CellProfiler software and Ilastik toolkit, which takes advantage of machine learning algorithms. This method allowed for a thorough analysis of big image sets and reduced both times of analysis and human bias in data processing (Figure 6A). After automated segmentation of EB3 foci with Ilastik, the CellProfiler pipeline was used to measure different parameters of size and shape of EB3 foci located underneath AChR clusters. We found that area, perimeter, and maximal and minimal Feret diameter of EB3 foci were decreased in BTP2-treated myotubes (Figure 6B). To account for the limitations in the automated identification of EB3 puncta, we verified these results by manually measuring the surface of EB3 foci on a smaller data set using ZEN software. The analysis confirmed that the surface of EB3-rich areas under AChR clusters was decreased after BTP2 treatment (Supplementary Figure S3A). Since drebrin bundles actin filaments and couples them to microtubules, it was plausible that with blocked drebrin/actin binding there were fewer microtubules that locally coalesce [67]. To test that, we measured the average size of a single EB3 focus with the ZEN software and quantified the frequency of single and coalescent EB3 foci under AChR clusters (see Materials and Methods, Section 4.3). We found that there were fewer coalescent EB3 puncta after BTP2 treatment (Figure 6C). Altogether, our results show that drebrin plays an important role in the microtubule capture at the AChR clusters, which is crucial for their assembly.

### 2.5. Cell Surface Delivery of AChRs Is Not Affected by Drebrin Knockdown

**Figure 7:**
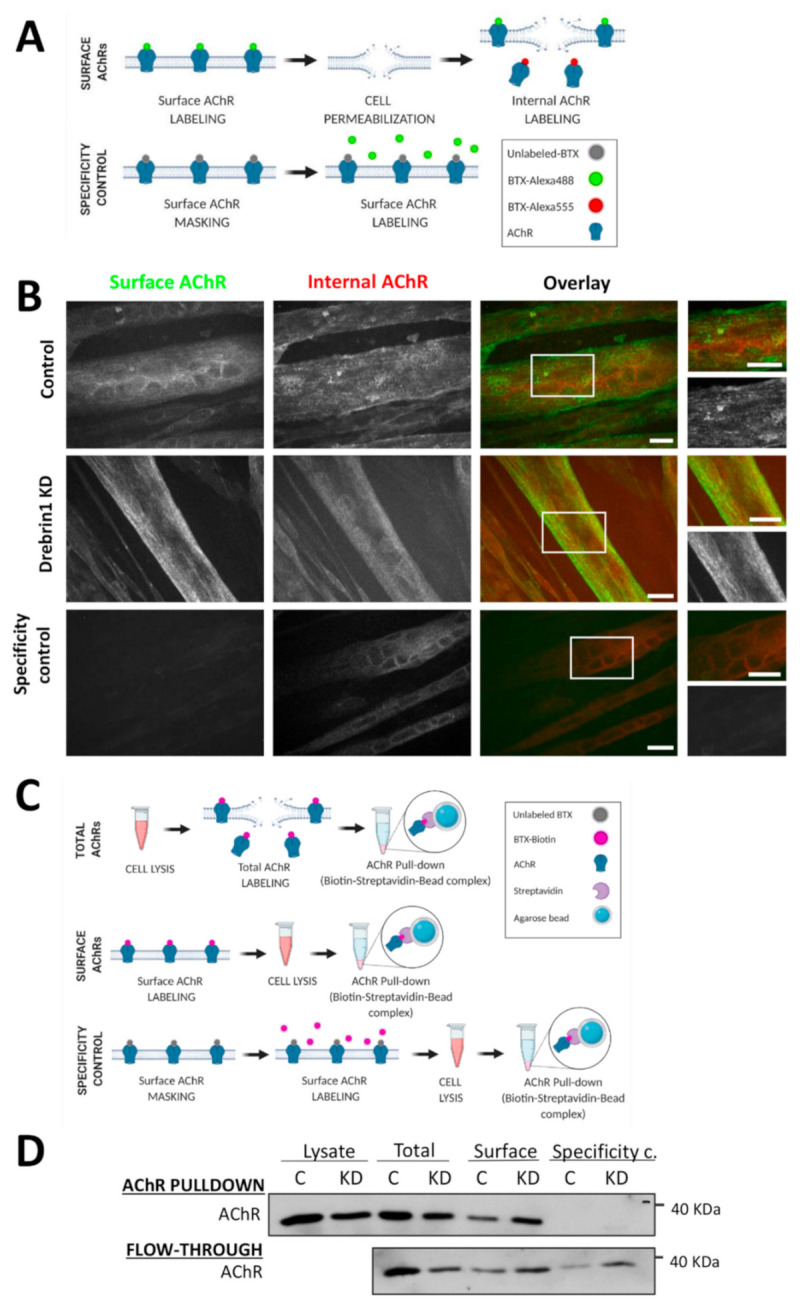
Cell surface delivery of AChRs is not affected by drebrin knockdown. (**A**) Schematic illustration of labeling surface and internal AChRs. (**B**) Surface AChRs (green) and internal AChRs (red) labeled with BTX-488 and BTX-555, respectively, in drebrin-depleted and control C2C12 myotubes transfected with non-targeting siRNA. Specificity control was performed by saturating myotubes with unlabeled BTX and subsequent labeling with BTX-488. Scale bars—20 μm. (**C**) Schematic illustration of AChR pull-down from drebrin-depleted and control myotubes. Specificity control was performed by saturating myotubes with unlabeled BTX and subsequent labeling with BTX-Biotin. (**D**) Pull-down of surface and total AChRs from the lysates of C2C12 myotubes after drebrin knockdown. AChR pulldown—immunoprecipitation of total and surface AChRs with BTX-Biotin and NeutrAvidin agarose beads. Flow-through—lysate after BTX-Biotin-NeutrAvidin complex pull-down. C—control myotubes transfected with non-targeting siRNA, KD—myotubes transfected with Dbn1-siRNA1. Illustrations were created with BioRender.com.

To further explore the mechanism through which drebrin impacts AChR organization, we assessed whether AChR delivery to the cell membrane depends on the expression of drebrin. To that end, we labeled surface and internal AChRs before and after permeabilization of C2C12 myotubes, respectively (Figure 7A,B). Surface receptors were labeled with BTX-488 and internal AChRs with BTX-555. A specificity control was performed by ‘masking’ surface AChRs with saturating concentration of unlabeled BTX and, after washing, incubating the myotubes with BTX-488 to label surface receptors. As anticipated, labeling of surface receptors was blocked by the incubation with unlabeled BTX (Figure 7B), confirming the specificity of the method. The BTX-488 signal was detected at the surface of both control and drebrin-depleted myotubes, suggesting robust delivery of AChRs to the cell membrane upon drebrin knockdown (Figure 7B). This result was additionally confirmed by pulldown of surface AChR from the myotubes. AChRs were bound to BTX-biotin incubated with cell lysates (total AChRs) or living cells (surface AChRs) (Figure 7C). Next, BTX-biotin-bound AChRs were precipitated from the lysates with NeutrAvidin beads. The specificity of the pulldown was confirmed by the absence of precipitated AChRs with unconjugated BTX (Figure 7D). In accordance with the result of immunolabeling, we observed no decrease in the surface AChR levels in drebrin-depleted myotubes (Figure 7D). Together, our results show that the impaired formation of postsynaptic machinery after drebrin knockdown is not a result of a hindered AChR delivery to the cell surface.

### 2.6. Drebrin Cooperates with AChR Clustering Regulator Rapsyn

**Figure 8:**
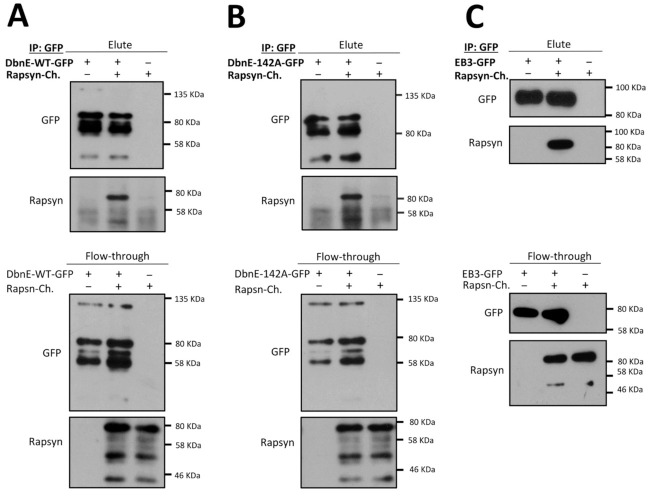
AChR-clustering regulator rapsyn interacts with drebrin. (**A**) Coimmunoprecipitation of overexpressed wild-type drebrin (DbnE-WT-GFP) with rapsyn (Rapsyn-mCherry) in HEK293 cells. (**B**) Coimmunoprecipitation of overexpressed phospho-dead drebrin mutant at Ser142 (DbnE-142A-GFP) with rapsyn (Rapsyn-mCherry) in HEK293 cells. (**C**) Coimmunoprecipitation of overexpressed EB3-GFP with rapsyn (Rapsyn-mCherry) in HEK293 cells. Elute—eluates from the beads incubated with equal volumes of lysates, Flow-through (FT)—lysates incubated with the beads.

Actin-organizing proteins such as MACF1 and actin cross-linker α-actinin were shown to bind to rapsyn, a scaffold protein crucial for the formation and maintenance of postsynaptic machinery [25,42,75,76]. MACF1 is also an organizer for the microtubule-associated proteins, such as EB1, and plays an important role in the recruitment of the MTs to the postsynaptic membrane [25]. Since MT capture at postsynaptic machinery was affected after drebrin knockdown, we decided to test whether drebrin and rapsyn interact. As shown in Figure 8A, overexpressed rapsyn indeed co-precipitated with drebrin from HEK293 cell lysate.

We also evaluated whether drebrin–rapsyn interaction depends on the phosphorylation of the S142 residue as it was shown for drebrin and EB3 [67]. Overexpressed phospho-dead drebrin mutant S142A was able to co-precipitate with rapsyn, suggesting that phosphorylation in this residue is dispensable for drebrin interaction with rapsyn (Figure 8B). Since AChR aggregation into clusters is regulated by the microtubule network, and we have observed aberrated MT attachment in the cluster area after drebrin-knockdown (Figure 5B,C), we decided to also check the binding of +TIP protein EB3 to rapsyn [25,40,41]. We found that similar to drebrin, its interactor EB3 also co-precipitates with rapsyn (Figure 8C).

**Figure 9:**
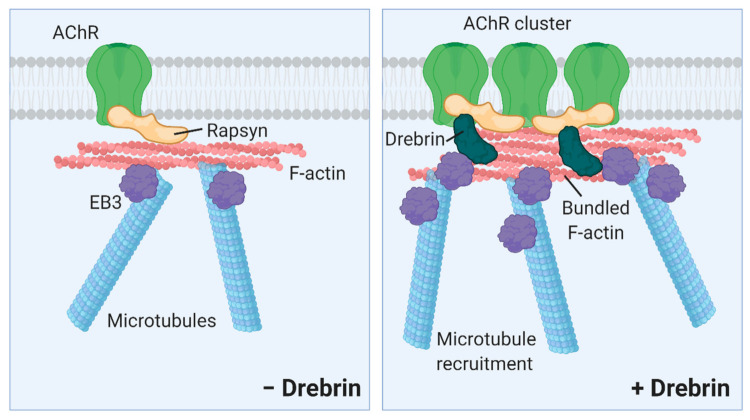
Proposed model of drebrin role in the regulation of postsynaptic machinery clustering. Rapsyn anchors AChRs to actin and microtubules (− Drebrin). Drebrin bundles actin filaments and acts as a cross-linker between actin cytoskeleton and microtubule network under AChR clusters. Drebrin interacts with rapsyn, a cluster-stabilizing protein, and EB3, a microtubule plus-end protein, and recruits microtubules under AChR clusters (+ Drebrin). Illustration created with BioRender.com (www.biorender.com, last access 26 August 2021).

These results support our conclusion that inhibited AChR cluster formation observed in drebrin-depleted myotubes is rather due to aberrant AChR aggregation possibly regulated by rapsyn and not due to impaired recruitment of the receptors to the myotube surface. Our findings provide a novel insight into the regulation of postsynaptic machinery clustering by showing that actin and microtubule cross-linker drebrin, plus-end-tracking protein EB3, and scaffolding protein rapsyn may act in concert to regulate AChR assembly (Figure 9).

## 3. Discussion

Various actin-organizing proteins have been implicated in the postsynaptic machinery organization and development of neuromuscular disorders [29,77,78]. Recent studies provide more evidence on the importance of proteins regulating both actin and microtubule cytoskeleton in the organization of the postsynaptic apparatus at NMJs [25,35,40]. Therefore, we have decided to test the role of drebrin, the actin and MT cross-linker, in the organization of AChRs. Drebrin has a well-established role on the synapses in CNS, but the function of this protein at NMJs remains unclear.

We identified drebrin as a novel postsynaptic protein at NMJs colocalizing with AChRs both in vitro and in vivo independently of the myofiber type (Figure 1, Figure 2 and Figure 3). We also show that, in myotubes, drebrin localizes to perforations in AChR clusters and overlaps with the condensed F-actin of synaptic podosomes (Figure 3). Enrichment of drebrin at podosome-like structures was previously reported in neuronal and non-neuronal cells, but not in myotubes [51,52,54]. Similar to drebrin, another actin and MT organizer, MACF1, localizes to AChRs at NMJs and to synaptic podosomes in cultured myotubes [25]. Drebrin transition between different components of the cytoskeleton is an important molecular mechanism underlying neuronal migration and contributing to the development of neuronal disorders [44,47,79,80]. In a similar manner, drebrin can be recruited to different cytoskeletal components at NMJs, where it links actin and rapsyn, and regulates MT reorganization. Such transition from the initial binding to MTs to further attachment to rapsyn during the ‘plaque-to-pretzel’ remodeling has been already proposed for MACF1 [25]. Detailed studies are necessary to assess the dynamic changes in drebrin localization at the postsynaptic machinery and to identify the potential protein partners regulating drebrin function at the different parts of the cytoskeleton.

Drebrin phosphorylation by Cdk5 at the S142 residue of the coiled coil (CC) domain triggers its actin-bundling activity and the interaction with EB3 protein, allowing drebrin to coordinate actin filaments and MTs at the same time [67,81]. Importantly, Cdk5 plays a key role at NMJs in the dispersal of AChRs in response to acetylcholine [82,83]. Thus, we verified whether drebrin localization at NMJs depends on the S142 residue, known to be phosphorylated by Cdk5. Since drebrin phospho-dead mutant S142A still localizes to AChRs, drebrin is most likely recruited to NMJs independently of the phosphorylation of S142 and EB3 binding (Figure 2). One of the other possible candidates that might regulate drebrin function at NMJs are Homers, scaffold adaptor proteins mediating multiple interactions at the postsynaptic density in the CNS [84,85]. Drebrin binds to Homer with the C-terminal motif PPXXF and Homer tetramer regulates actin bundling by drebrin [86,87,88]. Importantly, Salanova and colleagues found that Homer is enriched at the postsynaptic machinery of human NMJs [89,90]. Homer also binds to Cdc42 GTPase, an important cytoskeletal organizer of the postsynaptic apparatus [88,91,92]. It would be interesting to further explore what potential interactions regulate drebrin function at NMJs and whether Homer proteins are coordinating this molecular crosstalk.

Our study shows that drebrin is involved in the organization of postsynaptic machinery. Upon drebrin knockdown, the formation of AChR clusters was significantly diminished (Figure 4). We observed this decrease in the case of both total and complex AChR clusters (Figure 4C–G). We obtained a similar phenotype when drebrin’s binding to actin was blocked with the BTP2 inhibitor (Figure 4J,K). Since we have detected drebrin at AChR-occupied areas of clusters and in the perforations at synaptic podosomes, it is plausible that drebrin might regulate not only AChR clustering but also maturation. Indeed, we observed a decrease in the number of active podosomes, highlighting the possible role of drebrin in the maturation of the postsynaptic machinery (Figure 4H,I).

The impaired formation of clusters might be caused by the decreased expression of postsynaptic proteins, their impaired recruitment to the postsynaptic membrane, and/or defects in the assembly of clusters. Since we have not found consistent changes in the expression or in the delivery of postsynaptic proteins to the myotube surface, we focused on the role of drebrin in AChR clustering (Figure 7 and Supplementary Figure S2). Previous studies revealed that MTs play an important role in the incorporation of the new AChRs to the pre-existing clusters but they are not crucial for their maintenance [38,39]. Indeed, we found that upon drebrin knockdown, the number of MT plus-ends labeled with EB3 was decreased under AChR clusters (Figure 5B,C). Disrupted MT organization at the postsynaptic machinery might be caused by alterations in MT capture or stability. However, the number of EB3 foci remained unchanged outside the area of AChR clusters after drebrin knockdown or BTP2 treatment (Supplementary Figure S3B,C), indicating that the observed phenotype was a result of impaired MT capture specifically under the clusters and not overall stability. After the treatment with BTP2, there were fewer areas with accumulated EB3-positive puncta under AChR clusters, but the number of EB3 foci was comparable to the control (Figure 5D,E). This could be explained by the fact that silencing drebrin expression more broadly alters the signaling pathways regulating the organization of the cytoskeleton, whereas specific inhibition of actin-binding by drebrin results in a more limited mechanistic effect on the cytoskeleton. Drebrin bundles actin filaments and couples MTs to them, which might explain the decrease in the accumulated EB3 foci under clusters when drebrin interaction with actin is inhibited [67].

In line with previous studies, our results emphasize the role of MT capture at the AChR clusters and suggest the involvement of drebrin and EB3 in that process. Plus-end-tracking protein CLASP2 and its interactor LL5β also impact AChR distribution at the postsynaptic membrane by regulating the distribution of microtubules [37,38,93]. AChR clustering and the attachment of cortical MTs in myotubes are also regulated by the multi-scaffold protein liprin-alpha-1, which binds to the dystrophin-associated glycoprotein complex [39]. Microtubule reorganization by CLASP2 is agrin-dependent and the localization of liprin-alpha-1 at NMJs relies on the signal from the nerve [37,39]. We show that drebrin downregulation affects the formation of AChR clusters both in agrin- and laminin-induced models, the latter being devoid of nerve-derived signals, suggesting that drebrin, liprin, and CLASP2 pathways are not redundant (Figure 4C–G). Nevertheless, in-depth studies are required to assess the relationship between the pathways controlled by those actin and MT organizers.

Rapsyn governs AChR clustering at the postsynaptic apparatus, where it anchors receptors to the cell membrane and to the actin and microtubule network [94]. However, the molecular interactions of rapsyn with cytoskeletal partners are still poorly understood. Another hint that drebrin is involved in the assembly of clusters at the postsynaptic membrane is that both drebrin and its interactor, EB3, bind rapsyn (Figure 8A-C). It was proposed that the dispersal of AChRs is regulated through the interaction of α- and β-catenin with rapsyn, which provides a link between synaptic receptors and actin cytoskeleton [95]. We show that drebrin and EB3 interact with rapsyn, which might provide a cytoskeletal connection for the AChRs important for the cluster assembly (Figure 9). Moreover, the Wnt3a/beta-catenin pathway represses rapsyn expression and triggers the disassembly of AChR clusters both in vitro and in vivo [96]. Though we have observed some changes in rapsyn expression levels, rapsyn expression was not consistently affected by drebrin knockdown (Supplementary Figure S2D).

On the other hand, drebrin might cooperate with other actin organizers. How might drebrin regulate the function of proteins, such as beta-catenin? It is possible that when drebrin association with actin is changed, it impacts the interactions of other actin-binding proteins. It was shown that drebrin competes for binding to F-actin with cofilin and α-actinin, and inhibits the actin filament severing and cross-linking, respectively [97,98,99]. Drebrin downregulation might result in increased actin severing by cofilin and release actin-bound proteins, such as beta-catenin, enabling its translocation to the nucleus. Moreover, changes in the conformation of actin filaments caused by drebrin modify the preferential binding of other actin-organizing proteins to different actin cytoskeletal structures [100,101]. Another possibility is that drebrin silencing interferes with α-actinin and rapsyn interaction. A-actinin forms a ternary complex with rapsyn and AChRs, and its knockdown inhibits AChR clustering [42]. It was proposed that α-actinin and rapsyn binding might be regulated by the Cdk5, which also phosphorylates drebrin [42,66,67]. It is noteworthy that drebrin colocalized with α-actinin in sarcomeres (Supplementary Figure S1), but the relationship between these two proteins at NMJs requires further investigation.

In conclusion, our data indicate that drebrin might affect AChR clustering through rapsyn binding and/or by modulating the binding of other cytoskeletal organizers to actin. In future studies, it would be valuable to analyze the molecular interconnections regulating drebrin and rapsyn interaction, and their role in the organization of the postsynaptic machinery.

## 4. Materials and Methods

### 4.1. Cell Culture

C2C12 cells were purchased from the European Collection of Authenticated Cell Cultures (91031101, Merck, USA) and cultured for up to five passages in Dulbecco’s Modified Eagle’s Medium (DMEM), supplemented with 20% foetal bovine serum (FBS), 4.5 g/L glucose, L-glutamine, penicillin, streptomycin, and fungizone on 0.2% gelatin-coated plastic dishes. To allow the cells to form complex AChR clusters slides were covered with 2 μg laminin 111 (L2020, Merck) in 200 µL of DMEM per well and incubated for 24 h at 37 °C before cell seeding. Next, cells were trypsinized and plated on laminin-coated Permanox slides in eight-well Flexiperm chambers (94.6032.039, Sarstedt, Germany) or 48-well plates at seeding density of 1 × 10^5^ cells/well. For AChR pull-down experiment, cells were plated on 6-well plates at a seeding density of 1.5 million cells/well, and for co-immunoprecipitation experiments, cells were plated on 10 cm plates at a seeding density of 8.5 million cells/plate. For experiments involving agrin, cells were plated on Permanox slides coated with 0.2% gelatin. To induce cell fusion, 48 h after plating cells on laminin or gelatin, the medium was replaced with 2% horse serum-containing DMEM with 4.5 g/L glucose, L-glutamine, penicillin, streptomycin, and fungizone. On the fourth day of fusion, myotubes were stimulated with 10 nM agrin (550-AG-100/CF, Biokom) to form simple AChR clusters. The cells were harvested or fixed on the fifth day of fusion.

For inhibiting the drebrin-actin interaction, on 3rd day of fusion C2C12 myotubes were incubated with 5 μM BTP2 (N-[4-[3,5-bis(trifluoromethyl)pyrazol-1-yl]phenyl]-4-methylthiadiazole-5-carboxamide) (sc-221441, Santa Cruz Biotechnology, USA) diluted in dimethyl sulfoxide (DMSO) for 24 h [73]. After incubation cells were fixed on the 4-5th day of fusion. The control group (vehicle) was incubated with the same concentration of DMSO that the experimental group.

HEK293 cells (American Type Culture Collection (CRL-1573)) were cultured in DMEM with 10% FBS, 4.5 g/L glucose, L-glutamine, penicillin, streptomycin, and fungizone and plated on six-well plates covered with 0.2% gelatin at a density of 1.4 × 10^6^ cells/well. The cells were harvested on the third day of culture. The state of all cell lines cultured was regularly monitored with an optical microscope.

### 4.2. Cell and Tissue Labeling

Myotubes were fixed in 4% paraformaldehyde (PFA) at 37 °C for 10 min. or in absolute methanol at −20 °C for 10 min. and stained using AlexaFluor-488-congujated BTX (B13422, ThermoFisher Scientific, USA) at room temperature for 15 min. For labeling of surface AChRs in living cells, AlexaFluor 488-BTX conjugates were added to the culture media for 15 min. After cell membrane permeabilization with 0.5% Triton X-100 for 1 h, internal AChR staining was performed with AlexaFluor-555-conjugated BTX (B35451, ThermoFisher Scientific). For the control of binding specificity (mask) surface AChRs were masked with unlabelled-BTX (B1601, ThermoFisher Scientific) added to the culture media for 15 min. prior surface AChR labeling as described above. For immunolabeling, cells were incubated with blocking buffer [2% BSA (bovine serum albumin), 2% normal goat serum (NGS), 0.5% Triton X-100, and 0.02% sodium azide] for 30 min. to 4 h. Then, cells were incubated overnight with primary antibodies diluted 1:250–1:500 in blocking buffer. After three washes with phosphate buffered saline (PBS), cells were incubated for 1 h with secondary antibodies diluted 1:500–1:1000. F-actin at podosomes was stained with AlexaFluor-633 Phalloidin (A22284, ThermoFisher Scientific) for 15 min. at room temperature. Cells were mounted onto slides with Fluoromount mounting medium (F4680, Merck) containing 4′,6-diamidino-2-phenylindole (DAPI) (A4099, AppliChem, USA).

Skeletal muscle tissues (*tibialis anterior*, diaphragm, *soleus*) were isolated and fixed in 4% PFA for 20–70 min., depending on the muscle size or in methanol for 3–5 min. In the case of *tibialis anterior* and *soleus*, the entire hindlimb was dissected and muscle-covering fascias were removed before fixation. *Triangularis sterni* muscle was fixed by incubating the whole ribcage in absolute methanol at −20 °C for 3 min.

Whole mount preparations or teased muscle fibers were incubated in blocking buffer for 8 h or 30 min., respectively. Primary antibody incubation was performed at 4 °C overnight with rotation at concentrations 1:200–1:500. Next, fibers were washed three times with PBS and incubated with secondary antibodies at room temperature for 1.5 h. After three washes with PBS, fibers were incubated with AlexaFluor-488 BTX for 15 min. After final washes, specimens were mounted with Fluoromount mounting medium containing DAPI.

The primary antibodies were as follows: anti-drebrin (10260-1-AP, ProteinTech, and 25770-1-AP, ProteinTech), anti-phospho-drebrin (MABN833, Merck), anti-EB3 (612156, BD Biosciences, USA), anti-myosin heavy chain (AB_2147781, DSHB, USA), anti-α-actinin (sc-17829, Santa Cruz Biotechnology), anti-Ryr1 (ab2868, Abcam, USA), anti-neurofilament (AB_2314897, DSHB), and anti-synaptophysin 1 (101 004, Synaptic Systems, Germany). The secondary antibodies were: anti-rabbit AlexaFluor-568 (A-11004, ThermoFisher Scientific), anti-mouse AlexaFluor-568 (A-31570, ThermoFisher Scientific), anti-mouse AlexaFluor-488 (A28175, ThermoFisher Scientific), anti-mouse AlexaFluor-647 (A-21236, ThermoFisher Scientific), and anti-guinea pig AlexaFluor-647 (A-21450, ThermoFisher Scientific).

### 4.3. Microscopy and Image Analysis

Microscopic analysis was performed with Axio Observer Z.1 inverted microscope (Carl Zeiss, Germany) equipped with CSU-X1 spinning disc unit (Yokogawa, Japan), Evolve 512 EMCCD camera (Photometrics, Tucson, AZ, USA), and diode 405/488/561 and 639 nm lasers (Carl Zeiss, Oberkochen, Germany). The images were collected and analyzed using ZEN 3.1 software (Carl Zeiss) and processed with FijiJ distribution of ImageJ 1.51 h software [102] or Adobe Photoshop CS6 (Adobe, San Jose, CA, USA). BTX-labeled AChR clusters were quantified with the Events tool from the ZEN Software and clusters with at least two perforations were considered to be complex. The results were calculated as an average number of clusters and presented as a percentage of the control (non-targeting siRNA).

For the quantification of EB3 foci, the AChR cluster shape was defined as the region of interest (ROI) with the contour tool on the BTX channel of Z-stack pictures from the whole myotube depth. Based on previous findings, we set the Z level for quantification at 0.5 μm below the level of highest fluorescence detected in the BTX channel, where the EB3 fluorescent signal starts to gradually increase from the tip of dynamic microtubules [103,104]. The Events tool was used to quantify the EB3 foci within the AChR cluster area on the EB3-detection channel. Contrast parameters were adjusted following the same method for each picture. The results were calculated as the number of EB3 foci per area (um^2^) and presented as a percentage of control (non-targeting siRNA). For the analysis of the organization of EB3 foci, Ilastik 1.3.3.post3 (European Molecular Biology Laboratory, Heidelberg, Germany) [105] and CellProfiler 4.0.7 (Carpenter Lab, Broad Institute of Harvard and MIT, USA; www.cellprofiler.org) [106] open-source software was used. Images were segmented with Ilastik’s pixel segmentation algorithm into “EB3 foci” and “background” pixels. The algorithm was manually trained with a selection of 10 images from either the DMSO or BTP2 condition. After training, the segmentation algorithm was applied to the total set of images (n = 30 images/group from at least three independent replicates), resulting in a binary version of the original microscopy image containing pixels classified as “EB3 foci”. This segmented image was analyzed with CellProfiler, where foci were identified as objects and measured for size and shape parameters. In order to limit the measurements to the area underneath AChR clusters, a mask with the shape of the corresponding cluster was applied to the EB3 image using the MaskImage Module of CellProfiler. Statistical analysis of the parameters referring to the size and shape revealed that surface, diameter and perimeter were significantly different between the DMSO- and BTP2-treated groups.

To validate Cell Profiler’s automatic analysis, manual measurement of EB3 foci surface was performed with ZEN software on 10 images per group from at least 3 independent replicates, measuring 15 foci per image (*n* = 150 measurements/group). Coalescent vs. single EB3 foci classification was performed on the same dataset. The surface of 15 single foci was measured and the average was used as a threshold. Foci with a smaller or equal surface than the threshold were classified as single, while foci with a bigger surface than the threshold were classified as coalescent.

### 4.4. Transfection with siRNA and Plasmids

Three out of four drebrin—specific siRNAs that were tested inhibited total AChR cluster formation. siRNA3 was tested separately on a different batch of C2C12 cells. The two most efficient siRNAs were chosen for further analyses. Three days after fusion myotubes were transfected with 20 nM siRNAs (Qiagen) targeting *Dbn1*-encoding transcripts. The siRNA sequences were as follows: siRNA1 [Mm_Dbn1_2, SI00974722, CAGCAGAGTCTGGAAGCTGAA], siRNA2 [Mm_Dbn1_3, SI00974729, CAGGAGGAAGAGTTCGCCCAA], siRNA3 [Mm_Dbn1_1, SI00974715, CCCGTTGGTAGTTGAAACAAT]. Transfection with siRNAs was performed with Lipofectamine RNAiMAX (13778075, ThermoFisher Scientific) according to the manufacturer’s instructions. The negative control (non-targeting) siRNA was AllStars Negative Control (1027280, Qiagen). After 48 h, cells were either fixed with 4% PFA or absolute methanol or processed for Western blot or qPCR analysis.

Transfections of HEK293 cells were performed at 80% confluency with TransIT-LT1 Transfection Reagent (MIR 2304, Mirus Bio) according to the manufacturer’s instructions. The plasmids were as follows: pEGFP-C1-DbnE-WT and pEGFP-C1-DbnE-142A (provided by Prof. Hiroyuki Yamazaki), pEGFP-N1-EB3 [107], and pShuttleCMV-Rapsyn- mCherry. After 48 h, cells were lysed for co-immunoprecipitation. Each day of the culture the transfection efficiency was assessed with a fluorescence microscope.

### 4.5. Generation of Stable C2C12 Line Overexpressing EB3-GFP

The pSBbiPur-EB3-GFP plasmid was constructed by amplification of the EB3-GFP sequence from the EB3-GFP plasmid [107] using 5′ TATATAGGCCTCTGAGGCCATGGCCGTCAAT 3′ and 5′ TATATAGGCCTGACAGGCCTTACTTGTACAGC 3′ primers. It was subcloned into the pSBbi-Pur vector (a gift from Eric Kowarz, Addgene plasmid #60523, http://n2t.net/addgene:60523; RRID:Addgene_60523) using SphI-HF enzyme [74]. Cultured myotubes were transfected with pSBbiPur-EB3-GFP plasmid and pCMV(CAT)T7-SB100 (a gift from Zsuzsanna Izsvak, Addgene plasmid #34879, http://n2t.net/addgene:34879; RRID:Addgene_34879) in a 10:1 ratio [108]. pSBbi-RP empty vector (a gift from Eric Kowarz, Addgene plasmid #60513, http://n2t.net/addgene:60513; RRID:Addgene_60513) was used as the control plasmid to monitor the transfection efficiency [74]. The transfection was performed using Lipofectamine 2000 (11668-030, ThermoFisher Scientific) according to the manufacturer’s instructions. Twelve and 72 h after transfection, pSBbiPur-EB3-GFP-expressing cells were selected by adding puromycin at 10 μg/mL. Then, the cells were expanded, plated on 10 cm dishes, and differentiated into myotubes as described above. Six days after fusion induction, EB3-GFP-overexpressing myotubes were lysed for co-immunoprecipitation.

### 4.6. AChR Pull-Down and Co-Immunoprecipitation

For cell surface delivery experiments, precipitation of total and surface AChRs was performed with BTX-Biotin (B1196, ThermoFisher Scientific). For the pulldown of surface AChRs, BTX-Biotin was incubated with living cells at 37 °C for 15 min. and washed with PBS. For total AChRs, BTX-Biotin was incubated with lysed cells at room temperature for 15 min. For control of binding specificity (mask) surface AChRs were masked with unlabelled-BTX (B1601, ThermoFisher Scientific) added to the culture media for 15 min. prior to surface AChR labeling as described above. The cells were scraped off the dish and lysed in pull-down lysis buffer (100 mM HEPES pH 7.4, 150 mM NaCl, 5 mM NaF, 2 mM EDTA, 1 mM phenylmethylsulfonyl fluoride (PMSF), 1% Igepal CA-630 (NP-40), and cOmplete protease inhibitor cocktail (11697498001, Roche, Switzerland). To ensure total cell lysis, cells were incubated at 4 °C for 30 min. and then passed through a 25-gauge needle. The lysates were incubated with NeutrAvidin beads (29200, ThermoFisher Scientific) at 4 °C overnight with rotation. Beads were collected by centrifugation, washed three times with washing buffer (100 mM HEPES pH 7.4, 150 mM NaCl, 0.5% NP-40, 1 mM PMSF, and cOmplete protease inhibitor cocktail), resuspended in 2X Laemmli Sample Buffer (1610737EDU, BioRad, USA), and boiled at 95 °C for 10 min.

For co-immunoprecipitation of EB3 and drebrin, myotubes stably overexpressing EB3-GFP were scraped off the dish in ice-cold PBS, spun down and pellets were resuspended in lysis buffer [50 mM Tris-HCl pH 8.0, 150 mM NaCl, 0.1–1% Igepal CA-630, 50 mM NaH_2_PO_4_, 10% glycerol, and cOmplete protease inhibitor cocktail]. For drebrin and EB3 co-immunoprecipitation with rapsyn, HEK293 cells were washed with ice-cold PBS and scraped off the dish in lysis buffer. Next, cells were lysed at 4 °C for 45 min. during intensive mixing with glass beads to provide efficient cell lysis. To further mechanically disrupt the cells, lysates were passed through a 25-gauge needle and spun at 4 °C for 10 min. Collected supernatants were used for protein precipitation.

Dynabeads-Protein G (10003D, ThermoFisher Scientific) were washed four times with PBS containing 0.1% NP-40 and incubated at room temperature for 30–60 min. with 1 μg per sample of anti-GFP antibodies (GTX26673, GeneTex; A11120, ThermoFisher Scientific) diluted in 50 μL of PBS containing 0.1% NP-40. In the case of the control, IP beads were incubated with the buffer omitting the antibody. Next, the beads were washed with PBS containing 0.1% NP-40 and with lysis buffer and incubated with 80–200 μL of cell lysates at 4 °C overnight. For EB3-overexpressing myotubes after precipitating EB3-GFP, endogenous drebrin was co-immunoprecipitated from the lysate. For HEK293 cells, equal volumes of lysates with exogenous DbnE-WT-GFP or DbnE-142A-GFP or EB3-GFP were mixed with equal volumes of lysate with exogenous Rapsyn-mCherry. After collecting flow-throughs, beads were washed four times in lysis buffer. Next, beads were resuspended in 30–40 μL 2X Laemmli Sample Buffer and boiled at 95 °C for 10 min. Eluted supernatants were magnetically separated, collected, and resolved through SDS-polyacrylamide gel electrophoresis.

### 4.7. Western Blot

The proteins were transferred to a nitrocellulose membrane (66485, Pall Corporation, USA) using Trans Blot Turbo (1704270, Bio-Rad). Membranes were blocked in 10% non-fat milk in Tris buffered saline with Tween (TBST) at room temperature for 1 h and probed with primary antibodies diluted 1:1000-1:10 000 in 5% milk in TBST at 4 °C overnight. The primary antibodies were as follows: anti-drebrin (GTX12350, GeneTex; ab60933, Abcam), anti-GFP (GTX26673, Genetex; ab32146, Abcam), anti-AChR-α1, α3, α5 (838301, BioLegend), anti-rapsyn (ab156002, Abcam), anti-GAPDH (sc-25778, Santa Cruz) and anti-tubulin (ab18251, Abcam). After washing with TBST, the membranes were incubated with secondary antibodies conjugated to horseradish peroxidase [anti-rabbit HRP (111-035-144, Jackson Immuno Research, USA), anti-mouse HRP (7076, Cell Signaling, USA), anti-rat HRP (ADI-SAB-200-J, Enzo Life Sciences), and anti-goat HRP (sc-2020, Santa Cruz)]. Proteins were detected with Femto chemiluminiscent substrate (34095, ThermoFisher Scientific) and developed on X-ray films (771468, Carestream, USA).

### 4.8. Muscle Electroporation

Wild-type C57BL6 mice were obtained from Jackson Laboratories and maintained in the Animal Facility of the Nencki Institute of Experimental Biology, equipped with a selected pathogen free (SPF) barrier (standard). All experimental procedures were approved by the First Warsaw Local Ethics Committee for Animal Experimentation (629/2018). For muscle isolation, the animals were humanely killed by using a lethal dose of isoflurane or, in the case of pups, decapitation.

DNA electroporation was performed on P30-45 male mice under general anesthesia of ketamine/xylazine cocktail (150/10 mg/kg). After checking reflexes and shaving the hindlimb area to be injected, 25 μg DNA (1 μg/μL in PBS) of pEGFP-C1-DbnE-WT or pEGFP-C1-DbnE-142A plasmid was injected into the *tibialis anterior* muscle using a Hamilton syringe. Empty pEGFP-C1 vector (Clontech) was used as a negative control. The muscle was electroporated with 10 pulses of electrical current (180 V/cm) of 20 ms each at 1 s intervals using an ECM-830 electroporator (45-0052INT, BTX Harvard Apparatus, USA). Lidocaine was applied to the injection area immediately after electroporation. During the whole procedure, the animal’s eyes were covered with hydrating eye gel to prevent them from drying and the condition of the animals was monitored. After the procedure analgesics were administered and the condition of animals was monitored regularly in the subsequent days. Mice were sacrificed 7 days after the procedure.

### 4.9. RNA Isolation and Quantitative PCR

RNA was isolated with TRIsure Reagent (BIO-38032, Bioline) according to the manufacturer’s instructions. cDNA was generated from 1 µg of templates using the High Capacity cDNA Reverse Transcription Kit (4374966, Applied Biosystems, USA). RT-qPCR was performed in triplicates for each sample using the Step One Plus Real-Time PCR system (Thermo Fisher Scientific) and SYBR Green PCR Master Mix (4309155, Thermo Fisher Scientific). The housekeeping gene glyceraldehyde-3-phosphate dehydrogenase (*Gapdh*) was used to standardize the samples. Results were analyzed with Step One software v2.3 using the standard curve method. The following primers were used: Gapdh (5′-GGCCTTCCGTGTTCCTAC-3′ and 5′-TGTCATCATACTTGGCAGGTT-3′), Drebrin (5′-GACCCAGGCCAGCGAAG-3′ and 5′- CCCAGCAGGTGATGTCGATT-3′).

### 4.10. Statistical Analysis

Statistical analysis was performed using the IBM SPSS Statistics 22.0 (SPSS Inc., Chicago, IL, USA) or GraphPad Prism 7 software (GraphPad Software Inc., San Diego, CA, USA) and the results were calculated as mean ± SD or ± SEM. One to three extreme upper or lower values identified as outliers per experimental group were not included in the statistical analysis of the EB3 foci quantification after drebrin knockdown (i.e., values that fell outside of 1.5 times the interquartile range between quartiles 1 and 3). The exclusion of outliers did not change the observed phenotype or the statistical significance. Levene’s test was used to test the assumption of homogeneity of variance. The two-tailed Student’s *T*-test was used to analyze differences between experimental groups with interval data that followed a normal distribution. For data that did not follow a normal distribution, the non-parametric Mann-Whitney U test was used. The Chi-square test was used to analyze differences between experimental groups with nominal data (frequencies). Results were obtained from at least three independent experiments and considered statistically significant when *p* ≤ 0.05 (*). Obtained data were presented graphically with SigmaPlot 8.0 (Systat, Richmond, VA, USA) as a percentage of control if not stated differently.

## Data Availability

Presented data are available from the corresponding author upon reasonable request.

## Supplementary Materials

The following are available online at https://www.mdpi.com/article/10.3390/ijms22179387/s1, Figure S1: Drebrin localizes to the contractile machinery in muscle fibers, Figure S2: Evaluation of drebrin knockdown with siRNA3 and impact on rapsyn expression for all siRNAs. Figure S3: Control validations of the changes in microtubule organization after drebrin knockdown and BTP2 treatment.

## Abbreviations

AChR	acetylcholine receptors
BSA	bovine serum albumin
BTP2	3,5-bis-trifluoromethyl pyrazole
BTX	bungarotoxin
CLASP2	CLIP-associating protein 2
CNS	central nervous system
DGC	dystrophin-glycoprotein complex
DMEM	in Dulbecco’s modified Eagle’s medium
DMSO	dimethyl sulfoxide
EB1/EB3	end-binding protein 1 or 3
ECM	extracellular matrix
EGFP	Enhanced green fluorescent protein
ELK	ETS-like protein 1, ETS domain transcription factor 1
FBS	foetal bovine serum
GAPDH	glyceraldehyde-3-phosphate dehydrogenase
GEF	guanine nucleotide-exchange factor
HEK293	human embryonic kidney cells 293
LL5β	pleckstrin homology-like domain family B member 2
MACF1	microtubule-actin cross-linking factor 1
MT	microtubule
NGS	normal goat serum
NMJ	neuromuscular junctions
PBS	phosphate buffered saline
PFA	paraformaldehyde
PIP3	phosphatidylinositol-3,4,5-triphosphate
TBST	Tris buffered saline with Tween
TIP	plus-end-tracking proteins
WAVE1	WASP-family verprolin-homologous protein 1
