# Supplementary material for: Drebrin Regulates Acetylcholine Receptor Clustering and Organization of Microtubules at the Postsynaptic Machinery
Source: Int J Mol Sci. 2021 Aug 30;22(17):9387. doi: 10.3390/ijms22179387 (PMC8430516; doi:10.3390/ijms22179387)
Supplement: Supplementary file 1 [file ijms-22-09387-s001.zip › ijms-1339665-supplementary.pdf]

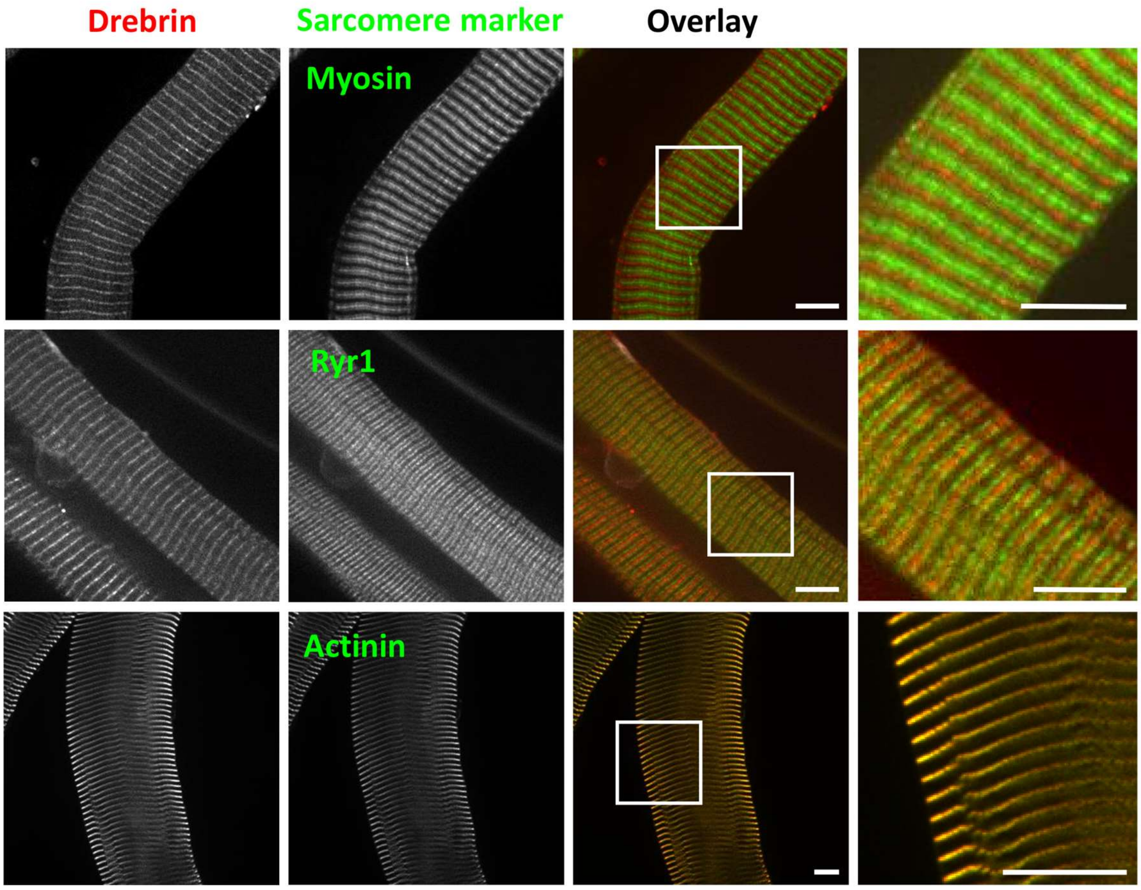

Figure S1. Drebrin localizes to the contractile machinery in muscle fibers. Colocalization of drebrin (red) with sarcomere markers, myosin, Ryr1 and actinin (green) in *triangularis sterni* muscle at P30. Scale bars - 10 μm. .

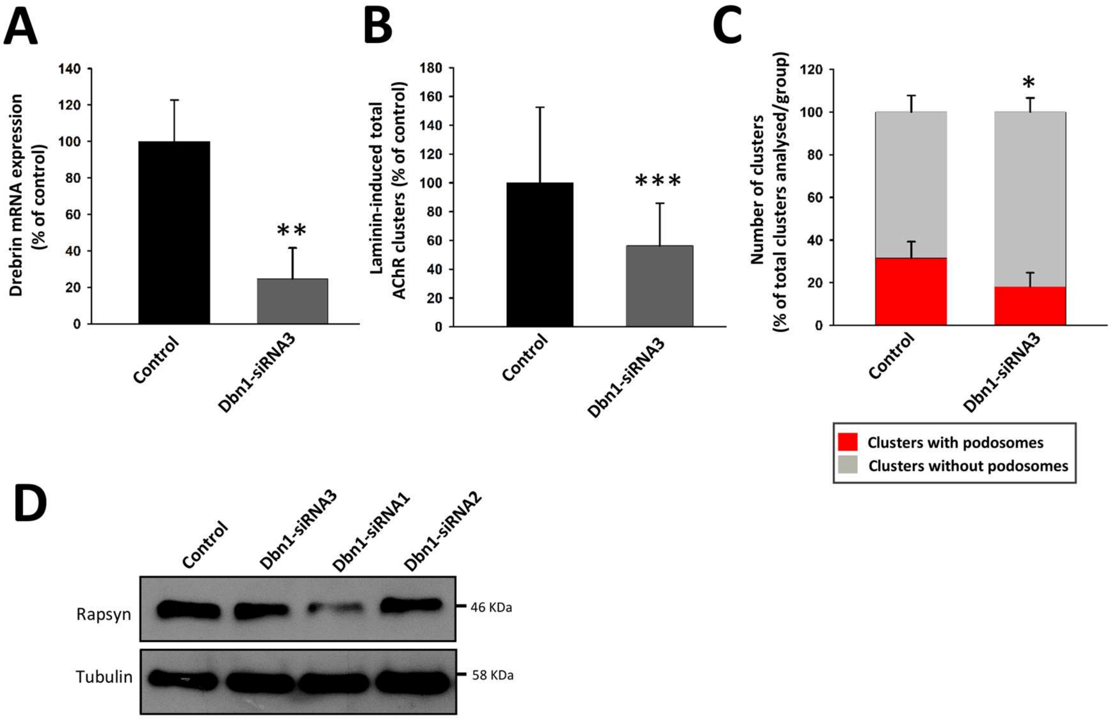

Figure S2. Evaluation of drebrin knockdown with siRNA3 and impact on rapsyn expression for all siRNAs**. (A)** Validation of Drebrin1 knockdown with siRNA3 (RT-qPCR). **(B)** Quantification of the number of laminin-induced total AChR clusters in drebrin-depleted myotubes (siRNA3). Data are presented as a percentage of control (myotubes transfected with non-targeting siRNA). **(C)** Quantification of the number of AChR clusters with synaptic podosomes in drebrin-depleted myotubes (siRNA3). Data are presented as a percentage of total clusters in each group. Error bars represent SD. Student's T test, $p \leq 0.05$ (*); $p \leq 0.01$ (**); $p \leq 0.001$ (***). **(D)** Rapsyn expression upon drebrin knockdown with siRNA1, 2 and 3. .

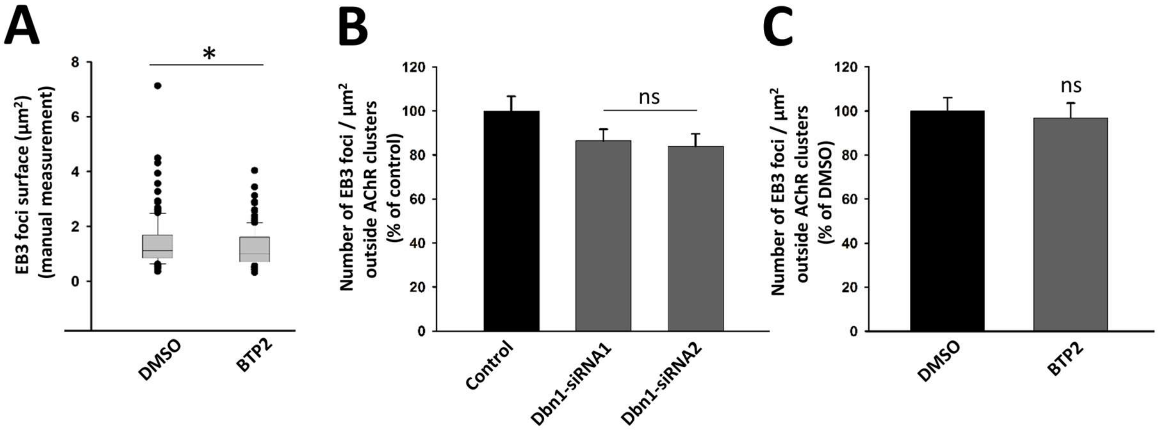

Figure S3. Control validations of the changes in microtubule organization after drebrin knockdown and BTP2 treatment. **(A)** Validation of automatic surface measurement of EB3 foci after BTP2 treatment. Manual measurement of the surface of EB3 foci was performed with ZEN software. Similarly to the automated measurement with CellProfiler, the surface of EB3 foci under AChR clusters was decreased in BTP2-treated myotubes. The box plots represent the distribution of the data: the bottom of the box represents the first quartile (Q1); the line within the box represents the median (Q2); the top of the box represents the third quartile (Q3); the whiskers (error bars) above and below the box indicate the 90th and 10th percentiles, respectively (values that fell outside are presented as black circles). Mann-Whitney U test; $p \leq 0.05$ (*). **(B)** Quantification of EB3 foci localized outside the AChR clusters area after drebrin knockdown or **(C)** after BTP2 treatment. Data are presented as a percentage of control (myotubes transfected with non-targeting siRNA). Error bars represent SEM. Student's T test, $p \geq 0.05$ (ns – not significant). .
